# The abdomen of *Drosophila*: does planar cell polarity orient the neurons of mechanosensory bristles?

**DOI:** 10.1186/1749-8104-3-12

**Published:** 2008-04-30

**Authors:** Caroline CG Fabre, José Casal, Peter A Lawrence

**Affiliations:** 1 Department of Zoology, Downing St, Cambridge CB2 3EJ, UK; 2 MRC Laboratory of Molecular Biology, Hills Rd, Cambridge CB2 0QH, UK; 3 Present Address: IBDML – Institut de Biologie du Développement de Marseille Luminy, UMR 6216, Case 907, Parc Scientifique de Luminy, 13288 Marseille Cedex 9, France

## Abstract

**Background:**

In the adult abdomen of *Drosophila*, the shafts of mechanosensory bristles point consistently from anterior to posterior. This is an example of planar cell polarity (PCP); some genes responsible for PCP have been identified. Each adult bristle is made by a clone of four cells, including the neuron that innervates it, but little is known as to how far the formation or positions of these cells depends on PCP. The neurons include a single dendrite and an axon; it is not known whether the orientation of these processes is influenced by PCP.

**Results:**

We describe the development of the abdominal mechanosensory bristles in detail. The division of the precursor cell gives two daughters, one (pIIa) divides to give rise to the bristle shaft and socket cell and the other (pIIb) generates the neuron, the sheath and the fifth cell. Although the bristles and their associated shaft and socket cells are consistently oriented, the positioning and behaviour of the neuron, the sheath and the fifth cell, as well as the orientation of the axons and the dendritic paths, depend on location. For example, in the anterior zone of the segment, the axons grow posteriorly, while in the posterior zone, they grow anteriorly. Manipulating the PCP genes can reverse bristle orientation, change the path taken by the dendrite and the position of the cell body of the neuron. However, the paths taken by the axon are not affected.

**Conclusion:**

PCP genes, such as *starry night *and *dachsous *orient the bristles and position the neuronal cell body and affect the shape of the dendrites. However, these PCP genes do not appear to change the paths followed by the sensory axons, which must, therefore, be polarised by other factors.

## Background

Developing animals are largely built from epithelial sheets. Within the plane of the sheet the cells may evince coordinated polarity — called planar cell polarity (PCP). For example, cells may be ciliated, with the cilia beating in a particular orientation, cells may move or divide with a biased orientation or they may produce structures that are themselves polarised. Clear examples are the oriented stereocilia made by the cochlear cells of the vertebrate inner ear [1-4], the strictly polarised hairs on a fly wing, each one being produced by a single epidermal cell [5,6], and the oriented growth of commissural axons towards the floor plate of the chicken embryo [7].

**Figure 1:**
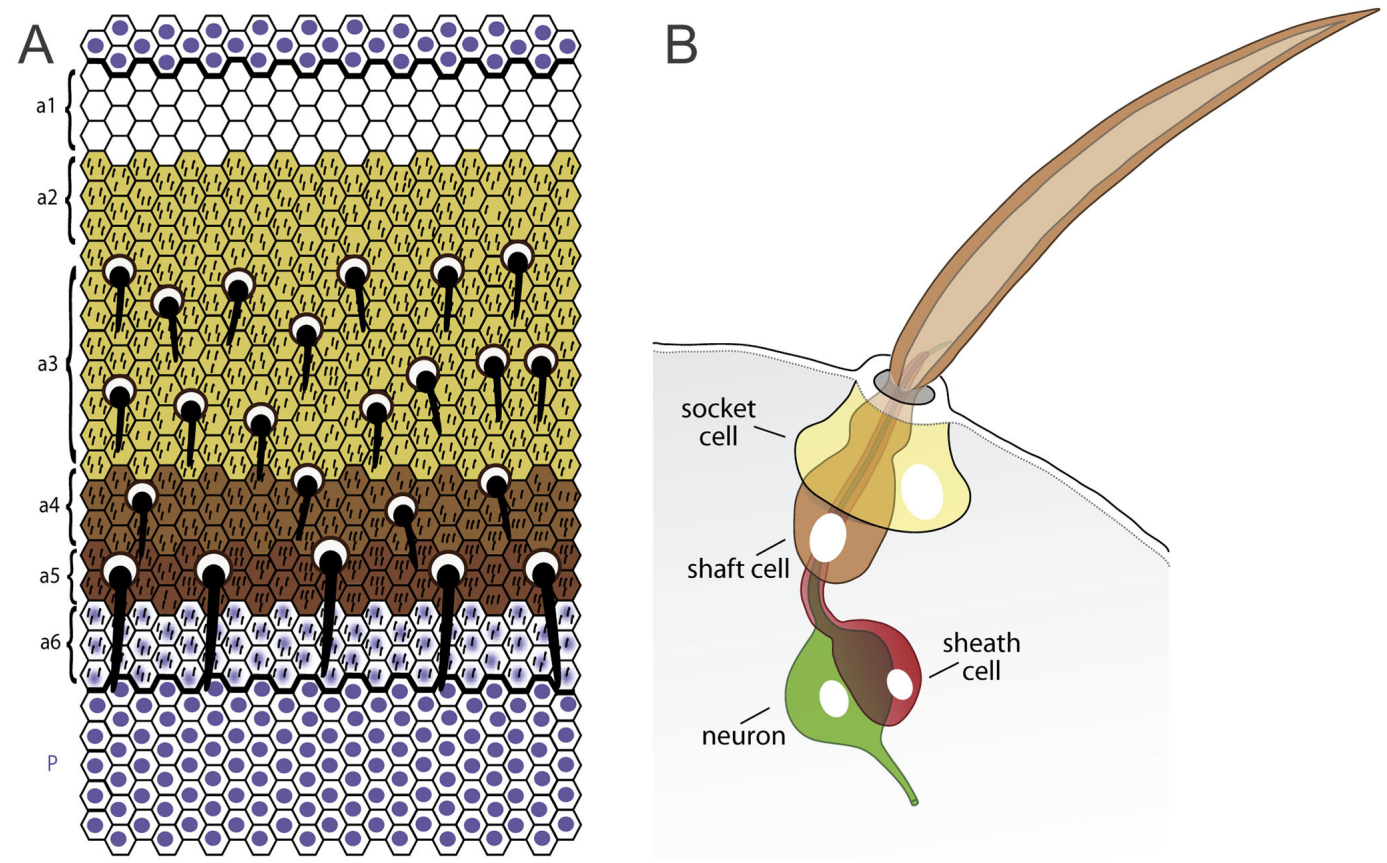
The abdomen and its mechanoreceptors.**(a) **The dorsal cuticle of the A compartment (cuticle types a3 to a5) contains oriented mechanosensory bristles, whereas a1, a2, a6 and the entire P compartment do not (after [8]). Anterior is up, posterior is down. **(b) **The four cells comprising the mature bristle are a shaft cell (brown), socket cell (yellow), a single-dendrite sensory neuron (green) and a glial-like sheath cell (red). The dendrite makes contact with the shaft at the dendritic cap [61]. As far as possible we use this colour code in the other figures.

The adult dorsal epidermis of the abdomen of *Drosophila *consists of a chain of alternating anterior (A) and posterior (P) compartments and produces a cuticle that displays a precise pattern of bristles and minute hairs (Figure 1a) [8]. Both the hairs and bristles originate from the histoblast nest cells that, during the pupal stage, propagate and migrate to replace the larval epidermis [9]. As they migrate, some of the epidermal cells in the A compartment become selected as sensory organ precursors (SOPs). Here, we find that in the abdomen, as in the thorax, each SOP then goes through a series of stereotyped asymmetric divisions [10-12] to generate first five and ultimately four cells that make a bristle (Figure 1b). These four cells are a clone and remain physically and functionally associated during bristle development. There are two external cells, the shaft cell that produces the long bristle shaft, and the socket cell that makes the socket surrounding the base of the shaft. There are two internal cells, the sheath cell, which wraps the neuron and presumably functions like the glia of vertebrates, and the mechanosensory neuron, whose dendrite attaches to the base of the shaft and whose axon extends to the central nervous system (CNS). The arrangement of these cells is polarised, as is the bristle that they produce.

In both the thorax and abdomen, bristles point posteriorly and this is largely due to the two genetic systems that build PCP [13-15]. It is advantageous that, like so many other organisms, the abdomen is metamerically segmented and also that the cellular events that make the mechanosensory bristles can be filmed. Using the abdomen, we ask if the genetic systems of PCP orient the dendrites and axons of the bristle neuron. To investigate, we used time-lapse confocal microscopy of living pupae as well as immunohistochemistry of dissected abdomens. We find that the sensory neurons are not all oriented in the anteroposterior (AP) axis; they behave consistently but differently in three regions of the A compartment (designated here as anterior, medial and posterior). The axons of all these bristles find their way to join the peripheral abdominal nerve that is located in the medial zone and they follow this nerve to the CNS. In each zone the mechanosensory neurons, their somata, dendrites and axons are polarised characteristically within the sensory cluster and with respect to the AP axis. The somata of the neurons move with respect to the bristles, moving laterally and posteriorly in the anterior zone and anteriorly in the posterior zone, movements that may involve nucleokinesis. Do these various aspects of polarity all depend on those PCP genes known to act on the orientation of the hair and shaft cells? We changed cell polarity by manipulating the PCP genes such as *frizzled *(*fz*) and *dachsous *(*ds*) [16] and found that the orientation of the shafts, the positioning of the neuronal cell bodies and the shape of the mechanosensory dendrites are indeed all influenced by PCP genes. However, these genes do not appear to orient the outgrowths or determine the pathways followed by the mechanosensory axons.

## Results

### Identification and development of the mechanosensory neurons

**Figure 2:**
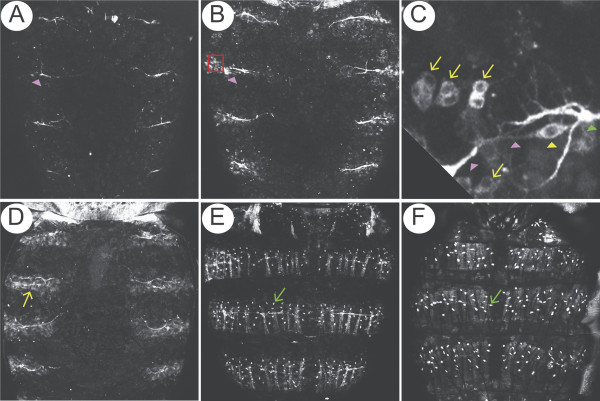
Development of mechanosensory neurons. Confocal projections of *elav.GAL4*, *UAS::cd8GFP *pupal abdomens showing segments A1-A4. **(a) **At 26.5 h APF, abdominal peripheral nerve (pink arrowhead) can be seen in each hemisegment. **(b) **At 29 h APF, cells expressing GFP have appeared laterally amongst the histoblasts that are migrating dorsally. The red square marks the area shown in detail in **(c)**: note the GFP-positive cell pairs oriented in the AP axis (yellow arrows), the peripheral abdominal nerve (pink arrowhead), a cell body of a md neuron [47] (yellow arrowhead) and a motorneuron axon (green arrowhead).**(d-f) **36 h APF (d), 47 h APF (e) and pharate adult (f); note the progressive elongation of the peripheral abdominal nerve towards the midline and the appearance of regularly-spaced cells that stain first weakly (yellow arrow) and then strongly (green arrows). As with all subsequent figures, anterior is up and posterior is down.

**Figure 3:**
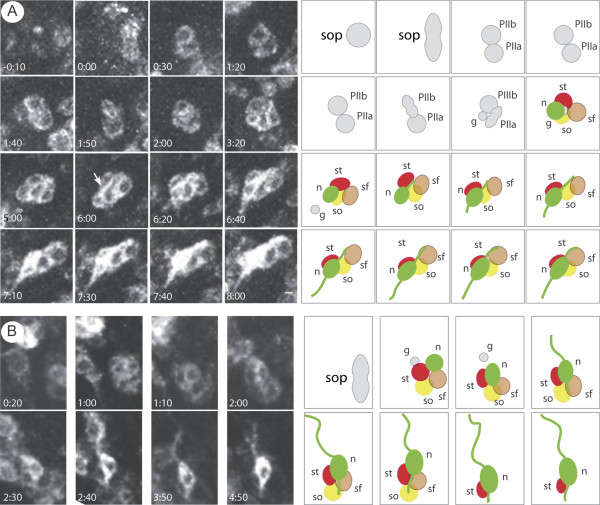
The differentiative divisions. **(a) **Divisions that generate a bristle in the anterior domain (nomenclature from reference [22]), anterior to top, dorsal midline to left. The left side shows frames from a film and the right side shows a representation, with colours code as in Figures 1b and 4; shaft cell (sf), brown; socket cell (so), yellow; neuron (n), green; sheath cell (st), red. The film starts at 27 h APF with the division of the SOP and is timed as in [22]. The first division is oriented close to the AP axis, and generates PIIb and PIIa. PIIa gives the shaft and socket cell, while PIIb divides to gives rise to PIIIb and a small fifth or glial cell that migrates away quickly and posteriorly (see 5.00). PIIIb makes the neuron and sheath cell. Later, 6–7 h from the first cell division, the dendrite (white arrow) and axon grow out in anterior and posterior directions, respectively. This sequence of events is similar to the notum apart from an inconsistency with respect to the fifth cell. We saw the fifth cell migrating away from the cluster well before the axons extended (Figure 3a, 5:00) while Gho and colleagues reported that the fifth cell migrated away along an axon [22], although their images do not show this axon; but Fichelson and Gho [62] reported that the fifth cell migrates away and undergoes apoptosis, apparently in the absence of an axon. In *Oncopeltus*, the fifth cell disappears before axon outgrowth [12]. **(b) **The same sequence is followed by SOP cells in the posterior domain, but the cell positions and orientations are different: the small fifth cell here migrates anteriorly, the dendrite and axon grow out in posterior and an anterior directions, respectively. See the scheme in Figure 4.

A membrane green fluorescent protein (GFP) fusion protein (*cd8::GFP*) was used to follow cell bodies, dendrites and axons in living pupae. *elav.Gal4 *was chosen to drive *UAS.cd8::GFP *[17]. However, although *elav *is best known as a marker of neuronal differentiation [18], it is also expressed earlier in the progenitor cells of the olfactory sensilla, some of which give rise to non-neural cells [19,20]. In the third abdominal segment, the first expression of GFP was seen at 27 hours after puparium formation (27 h APF) in a few cells that appeared laterally and near to the abdominal peripheral nerves (Figure 2a,b). These fluorescent cells did not resemble neurons and, by about 29 h APF, some were arranged in pairs amongst the histoblast cells (Figure 2b,c). These cells were followed by time-lapse confocal microscopy and they undertook a series of oriented asymmetric divisions (Figure 3a) as do dividing SOP cells in the thorax; we conclude that each pair of fluorescent cells is the product of the first division of an SOP. Taking one example from the anterior zone, this first division (Figure 3a at 0:00 h) was oriented more or less parallel to the AP axis [12,21] and gave rise to a pair of fluorescent cells (Figure 3a at 0:30). The more anterior cell (PIIb) divided next (Figure 3a at 1:50) to generate two daughter cells of different size, one larger PIIIb cell (Figure 3a at 2:00) and a smaller cell (Figure 3, 'g') that resembled in anatomy and behaviour the glial or fifth cell [12,22,23]. In all cases in the anterior zone, this cell quickly migrated away in the posterior direction (Figure 3a at 5:00). The more posterior cell (PIIa) divided to give rise to the socket ('so') and shaft ('sf') cells (Figure 3a at 3:20) [22]. The neuron ('n') and sheath cell ('st') were then generated by division of the PIIIb cell (Figure 3a. 2:00–5:00). The neuron became elongated and then formed a dendritic process (Figure 3a at 6:00) that extended towards the shaft cell and subsequently an axon grew out from the opposite side (Figure 3a at 6:40). While fluorescence increased within the neuron it decreased in the other bristle cells, almost disappearing by the end of the recording (Figure 3a at 8:00). Both dendrite and axon continued to elongate parallel to the AP axis but in opposite directions (Figure 3a at 6:00 to 8:00). A similar succession of events was seen in all the five *elav*-targeted fluorescent cells that were carefully followed by time-lapse microscopy. By 36 h APF there are many GFP-labelled clusters evenly dispersed within each hemisegment (Figure 2d–f).

SOPs in the posterior zone followed a similar series of divisions, but their polarity was not the same as in the anterior zone (see below, and Figure 3b). Also, there were differences in timing. Sensory cells in the anterior zone differentiated more slowly than those in the posterior zone; in the anterior zone, the axonal outgrowth began about 7 h after the first division (Figure 3a) while in the posterior zone some axons appeared after only 2 h 40 minutes (Figure 3b).

Incidentally, we noticed that some cells are phagocytosed in the pupa, cells that appear to be presumptive neurons because they express GFP under the *elav *promoter strongly, have elliptical shapes and produce short-lived bipolar protrusions (Additional file 1).

### The polarity of neurogenesis

**Figure 4:**
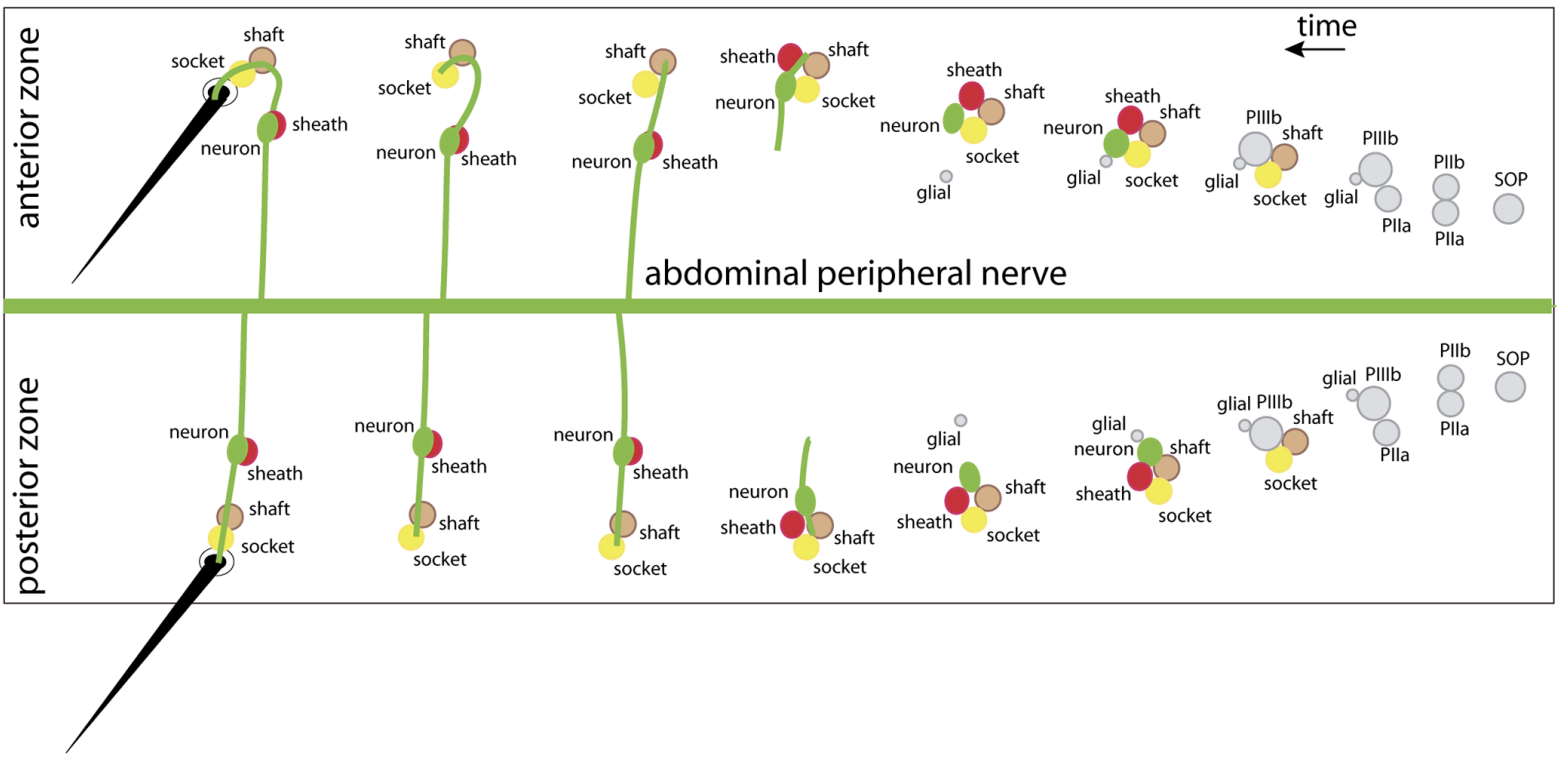
Polarisation and arrangement of the bristle cells in the two zones of the segment. A right hemisegment is shown with the mature bristles on the left, the earliest stages on the right and other stages in between. Most probably, the orientations of the divisions of the SOP and PIIa are the same everywhere, while the divisions of the PIIb cells are different in the two zones; for details, see the text and Figure 3. Considering the neurons (green) and their processes: in the anterior zone, initially the dendrite grows anteriorly, while in the posterior zone the dendrite grows posteriorly. The dendrite becomes enwrapped by the shaft and socket cells and, in the anterior zone, the tip of the dendrite then forms a U-shape in order to enter the socket cell (yellow). In the posterior zone the dendrite grows straight along the AP axis. In the anterior zone, the axon grows posteriorly while in the posterior zone it grows anteriorly. Note on this right hemisegment, the U-shaped dendrites turn to the left. On the left hemisegment the U-shaped dendrites would turn to the right. This summary model is based on our observations and on [22,61].

**Figure 5:**
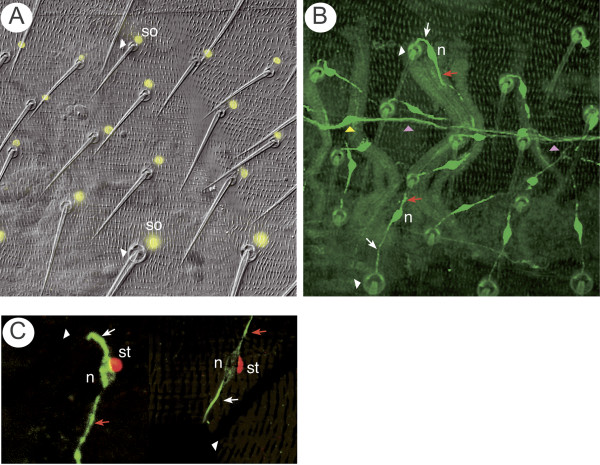
The dispositions of bristle cells in late pupae. **(a) **The socket cells (yellow nuclei) are marked by *Su(H).lacZ*; over the entire A compartment the nuclei are located lateroanterior to the socket (white arrowhead) of each bristle. **(b) **A right hemisegment to show the pattern of innervation of all the bristles, note the disposition of the cells and the axonal pathways depends on position (compare Figure 4). In the anterior zone, the dendrites (white arrows) make U-shaped turns between the sockets (white arrowheads) and the neuronal somata (n), and the axons (red arrows) extend posteriorly to meet the peripheral abdominal nerve (pink arrowhead). In the medial zone both axons and dendrites grow medially. In the posterior zone, the dendrites extend directly anteriorly from the bristles, and the axons continue anteriorly to join the nerve. The yellow arrowhead marks a md neuron that lies on the nerve [47]. **(c) **The sheath cell is marked by *pros.lacZ *and the neuron with 22C10. On the left the neuron is from the anterior zone and shows a U-shaped dendrite, with the axon growing posteriorly. On the right, a neuron is shown from the posterior zone, the neuronal soma is located anterior to the bristle and the axon grows anteriorly. In both cases the sheath cell (st) is closely associated with the neuron.

To help orient the reader, the development and final arrangements of all bristle and neuronal cells are shown in Figure 4 as well as the pattern of development of the progenitor cells. Note that there is a consistent AP polarity that orients the bristles and arranges the shaft and socket cells. However in the anterior and posterior zones of the compartment, the axons behave very differently, following pathways that form a mirror image pattern (Figure 5a). Also, the positions of progenitor cells vary with the zone of origin. For example, in the anterior zone (Figure 3a, 5:00) the fifth cell migrated in the posterior direction. In the posterior zone it migrated anteriorly (Figure 3b). Likewise, in the anterior zone, the neuron formed at the posterior edge of the sensory cluster and the sheath cell was formed anterior to the neuron. However, in the posterior zone, the neuron was generated at the anterior edge of the sensory cluster and the sheath cell localised posterior to the neuron (Figures 3 and 4). The other cells are thought to appear in the same positions in all sensory clusters throughout the A compartment: the socket cell being generated at the posterior edge of the cluster, and the shaft cell appearing anterior to the socket cell (Figure 4). Similar spatial and temporal variations were not found in the notum [22].

We followed the development of the fluorescent neurons in the living pupae up to the adult stage (Figures 2e,f and 5). In the pharate adult, each bristle is associated with a neuron with one dendrite (connected to the base of the shaft) and one axon (Figure 5b). During development, if an axon encounters another, it usually fasciculates with it and then continues on its way towards the abdominal peripheral nerve (Figure 5b) [24], eventually becoming bundled with that nerve. Neurons born in anterior and posterior zones behave differently: in the anterior zone the neurons protrude a dendrite from the anterior side and an axon from the posterior side of the soma, but in the posterior zone this is *vice versa *(Figures 3a,b, 4 and 5b). Most of the axons near the front and back of the A compartment elongate more or less parallel to the AP axis but axons emanating from bristles in the medial zone, that is, near to the abdominal peripheral nerve, grow laterally (Figure 5b). We also see three types of differently shaped and oriented dendrites: in the anterior zone the dendrites are strongly U-shaped, while in the posterior zone, the dendrites are straight and parallel to the AP axis. In the medial zone the dendrites are more or less parallel to the mediolateral axis (Figure 5b).

#### The somata of neurons in the three zones appear to show nucleokinesis.

**Figure 6:**
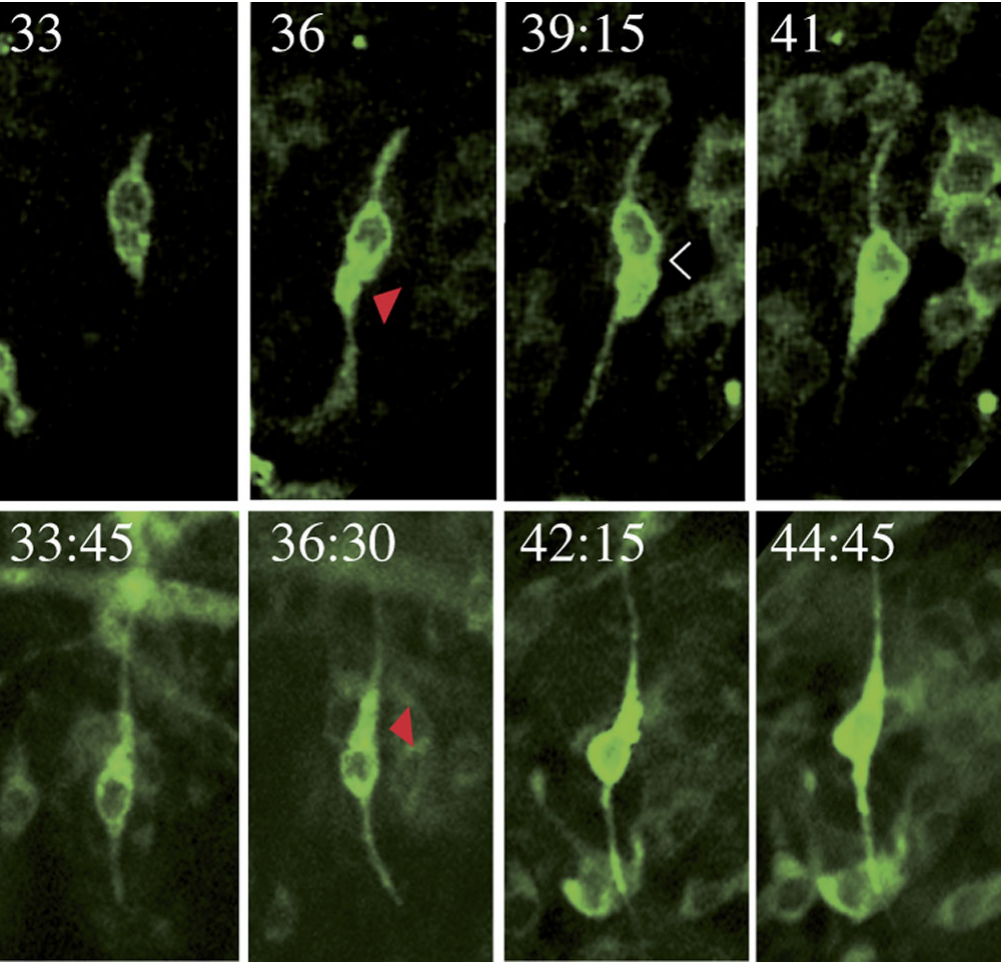
Time lapse observations of the dendrite elongating. Stills from two films (times given in hours and minutes APF), above an anterior zone bristle is shown, and below a posterior zone one; the stills are aligned with respect to the termination of the dendrites. The images show elongation of the dendrites. On the distal sides of the neuronal somata, in both cases, there are dilations (red arrowheads) and there are transient constrictions between the somata and the dilation (white arrowhead). Anterior, up; posterior, down.

In each zone, the cell bodies of the neurons become located differently with respect to the bristle they innervate. In the anterior zone the somata are usually positioned latero-posteriorly to their bristle (Figures 4 and 5b). While in the posterior zone, for both the smaller and larger bristles, the nuclei of their neurons are located anterior to the bristle (Figures 4 and 5b). Finally, the neuronal nuclei from medial zone bristles, localised around the area of the abdominal peripheral nerve, are positioned mainly laterally relative to the bristle (Figures 4 and 5b). How do they take up these positions? To answer this question, we followed the movement of the neuronal cell bodies in the pupa. While the axons are extending, large dilations are seen adjacent to the neuronal nucleus; these are always on the side of the axon and never on the side of the dendrite (Figure 6). These dilations appear to come and go and we see short-lived constrictions between the soma proper and the dilation. There are also transient swellings more distal to the soma. As the axon extends its tip, filopodia appear that are attached to the substrate and show searching movements. Associated with these events, the dendrite becomes longer. The distance between the soma and the tip of the dendrite increases (Figure 6). In vertebrate neurogenesis similar events are thought to be associated with neuronal migration by nucleokinesis [25,26].

**Figure 7:**
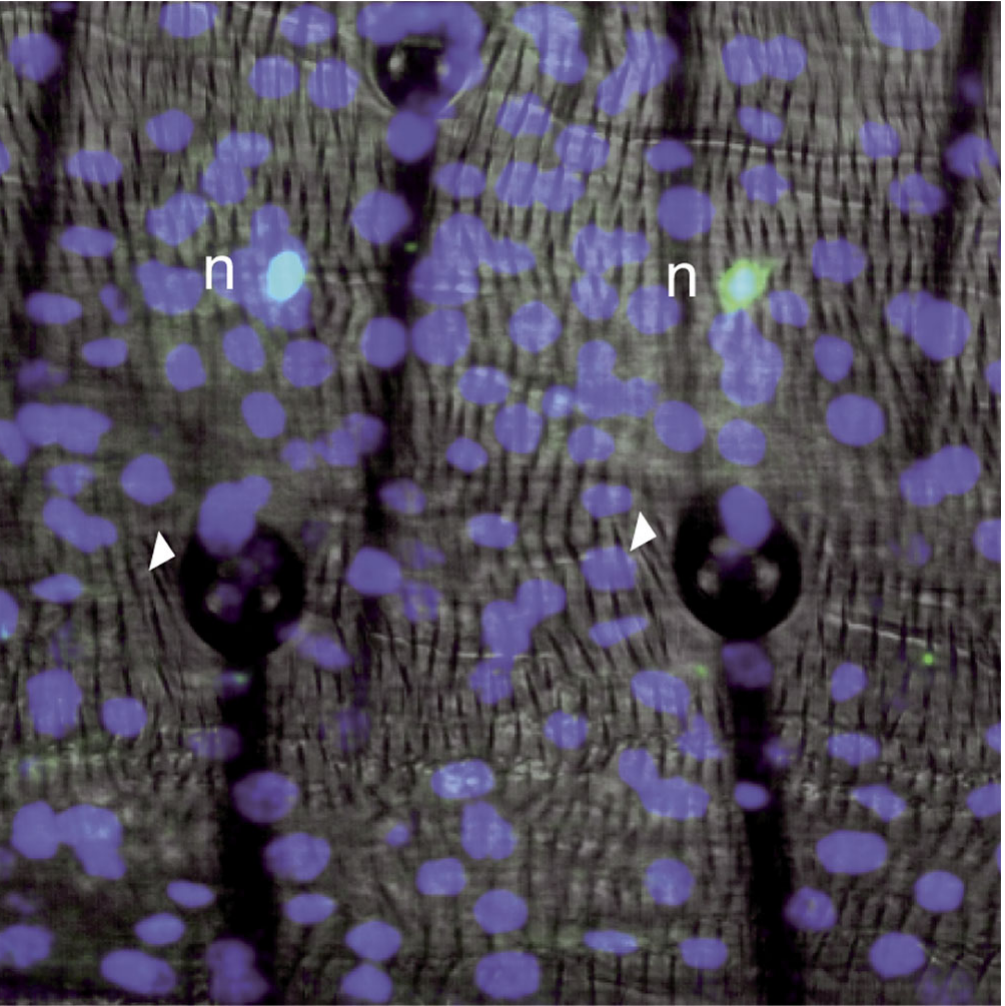
The location of the neuronal somata. The posterior zone of the anterior compartment of segment 3 showing two macrochaetes marked with the *his2::DGFP *construct and immunostained in the late pupa. Anti-Elav antibody identifies neuronal cell bodies (green) and anti-GFP marks the nuclei of all epidermal cells (blue). The nuclei of these neurons are located three to four epidermal cell widths away from their bristle.

We also observed somal movement in neurons in the posterior zone. The behaviour of both the cell body and the neuronal processes are as described above, except that the soma moved anteriorwards. In adult posterior macrochaetes, the distance between the nucleus of the neuron and the bristle socket can be as much as four epidermal cell nuclei (approximately 35 microns; Figure 7) and we suspect this separation is due, at least in part, to nucleokinesis.

### PCP and the polarisation of the mechanosensory neuron

In the wild type, all the bristle shafts point posteriorly and this is due to the two separate genetic systems responsible for PCP; one employs the *stan *and *fz *genes (the Stan system) and the other is built with the *ds *and *fat *genes (the Ds system) [14,27]. Thus, one gets the largest disturbances to PCP if both systems are knocked out, as in *ds*^-^*stan*^- ^flies. We now ask if either of these molecular systems polarise the dendrites and axons of the mechanosensory neurons. What is the effect on the axons and dendrites if the polarity of the bristle shafts and the nearby epidermal cells is altered? To answer these questions we used the PCP genes to change the polarity of the epidermis.

#### *UAS.fz*-expressing clones

**Figure 8:**
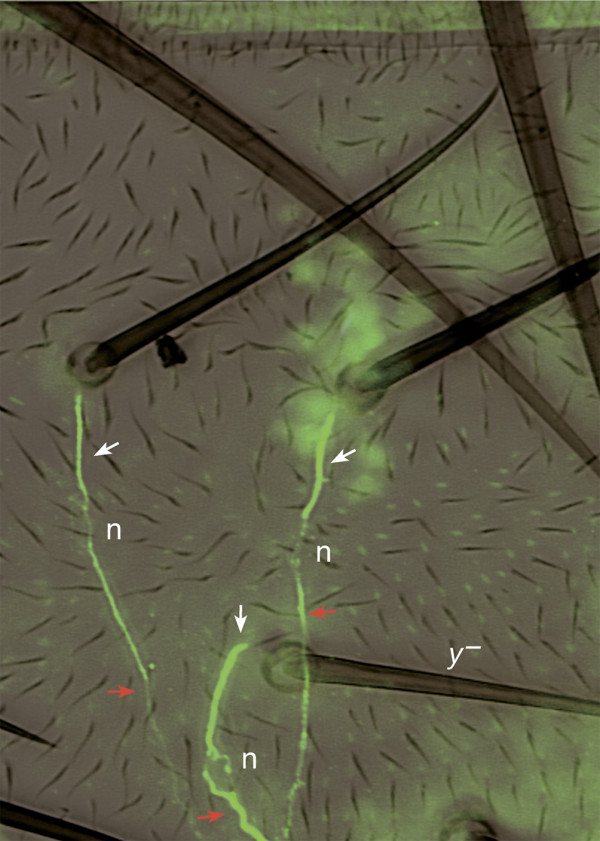
A *UAS.fz*-expressing clone affects bristle polarisation. Using young adults, neurons were stained with 22C10 antibody (green) and this image and a DIC image were combined. A *UAS.fz *clone was induced in the anterior domain of the A compartment. The clone is marked with the yellow marker that can be identified by the light colour of the bristles. Wild-type hairs and bristles that localize anterior to the yellow bristle (y^-^) have reversed polarity. These reversed bristles are localized in the anterior zone of the compartment, yet their dendrites (white arrows) are straight (no U-shape) and the somata of the neurons (n) are posterior to the bristles. The y^- ^bristle is also in the anterior zone but has lateral polarity; its dendrite (white arrow) makes a small U-shaped turn. Nevertheless, all axons (red arrow) grow posteriorly towards the nerve as they normally do.

Fz is required for normal cell polarity [28,29]. Clones of *UAS.fz-*expressing cells cause complete reversal of the polarity of the epidermal and bristle cells within the front of the clone and in the wild-type territory anterior to it (Figure 8) [30]. In the anterior zone of the wild-type fly the dendrites are U-shaped and the axons pursue a direct path posteriorly towards the peripheral abdominal nerve (Figures 5b and 8). However when, because of a nearby *UAS.fz-*expressing clone, a bristle is turned round to point anteriorly, the dendrite can and does take a straight path towards the bristle (no U-shape; n = 31). The neuronal cell bodies of these reversed bristles (microchaetes) are now found to be separated by two or three epidermal cells from the bristles (Figure 8), exactly the distance separating bristles (microchaetes) from the neural somata in the wild-type posterior zone. Whether a U-shape forms or not depends on the relative positions of the neuron and socket, and also of the orientation of the bristle.

It seems that the dendrite must enter the base of the shaft in a direction parallel to the axis of the shaft and if the neuronal body lies lateral or posterior to the bristle socket, the dendrite has to make a U-turn to do so. To test the contribution of the bristle shaft, we studied flies mutant for the *musashi *gene [31]. In such flies, the bristle shaft cell is frequently lost and there are, usually, extra socket cells. In these cases a U-shaped dendrite is never observed, the dendrite appearing to be connected directly to the socket cell (data not shown). This suggests that it is the entry to the base of the shaft that shapes the dendrite. Our observations show that overexpression of *fz *reverses the orientation of the dendrite as they extend from the soma but leaves the pathways followed by axons unaffected. The axons always follow their normal paths, whether the bristle and its neuron are overexpressing *fz *or are wild type or whether the epidermal cells amongst which it is growing are wild type or mutant.

#### *ectoDs*-expressing clones

These clones express a variant of the Ds molecule that lacks the cytoplasmic domain. They strongly reverse polarity of the bristles and epidermal cells behind the clone, a phenotype similar to that of *fz*^- ^clones [27]. However, clones expressing *ectoDs*, because they affect the Ds system, could alter polarity by a different mechanism than *fz*^- ^clones. Nevertheless, all aspects of bristle innervation are the same in both *fz*^- ^and *ectoDs*-expressing clones; for example, dendrites of reversed bristles located in the anterior zone no longer show a U-shape (n = 17; data not shown).

#### *ds*^- ^*stan*^- ^flies

**Figure 9:**
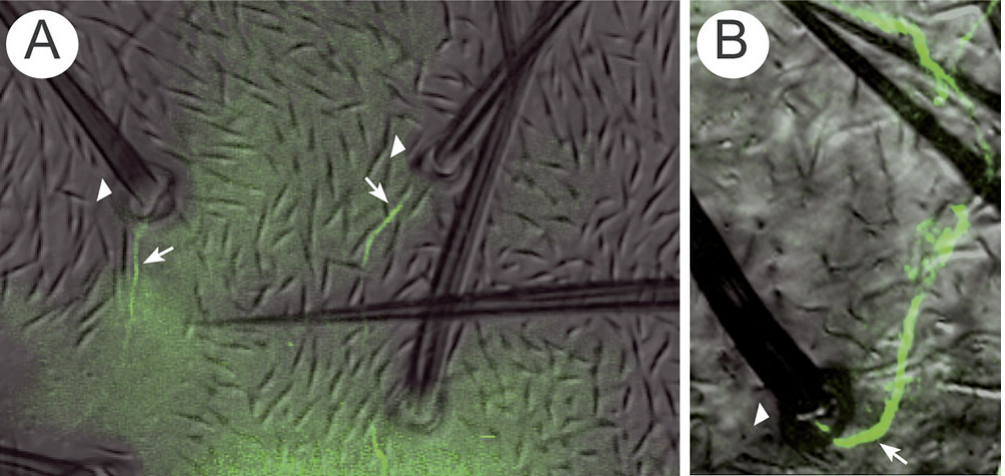
Polarity disturbance in *ds*^- ^*stan*^- ^pupae. Dissected *ds*^- ^*stan*^- ^late pupae stained for 22C10 for neurons. **(a) **Anterior zone of an A compartment. The hairs show disturbed polarity, bristles (sockets are marked with a white arrowhead) are reversed and their associated dendrites (white arrows) elongate straight with no U-shape. **(b) **Posterior zone of an A compartment showing a reversed bristle. Note that the dendrite (white arrow) has a U-shaped termination. Sockets are marked with white arrowheads.

When both the Stan and the Ds systems are defective, PCP is grossly disturbed, with the bristles and hairs pointing in many directions [27]. When individual bristles in the anterior zone are reversed, we see the same consequences as with *UAS*.*fz-*expressing clones; the U-shaped turns of the dendrites are absent (Figure 9a) and the neuronal somata are located posterior to the bristles. In the posterior zone, these *ds*^- ^*stan*^- ^pupae also have some reversed bristles, now connected to U-shaped dendrites (Figure 9b). Overall, the arrangements of bristles and neuronal somata are considerably disorganised in *ds*^- ^*stan*^- ^flies, as is clear from Figure 9. In wild-type abdomens about 60% of the neurons from the anterior zone lie lateroposterior to the bristles and 20% are posterior to them (n = 37; Additional file 2), while in *ds*^- ^*stan*^- ^flies only about 30% of these neurons are lateroposterior to the bristles and 60% are posterior (n = 37). However, in both anterior and posterior zones, once the axons have left the bristles, no matter how the bristles or epidermal cells are oriented, the axons follow routes typical of the wild type and grow directly towards the abdominal peripheral nerve near the middle of the A compartment.

## Discussion

We investigate, in the *Drosophila *abdomen, the contribution of PCP to the orientation of the neuronal components of the mechanosensory bristles, including the neuronal somata, axons and dendrites. We report that PCP has input into the form of the dendrites and the positioning of cell bodies. However, the pathways followed by axons appear to be constant, regardless of the orientation of the bristles and the PCP genotype. These axons also follow their usual paths, independent of the polarity (reversed or normal) of the epidermal cells they grow over.

### The development of abdominal bristles

The development of the adult bristles in the abdomen is not well known. Shirras and Couso [32] identified cells in the histoblast nests that stained for *neuralised *about 20 h APF, and suggested these might be precursors (SOPs) of the posterior row of macrochaetes. We see the first dividing SOP cells among the migrating histoblasts about 28 h APF, and the first signs of differentiating neurons some 90 minutes later (Figure 3a) before the shaft and socket cells differentiate [9]. At this time, the histoblast cells have reached only about half way on their journey to the dorsal midline [9]. It follows that the axons are able to orient towards the peripheral abdominal nerve, even as they are in the midst of a mass migration of epidermal cells.

In the larval sensory organs, and in most parts of the adult, including the wing margin and the notum, the sensory organs are generated close to the sites where they will finally differentiate [33]. In the notum, SOP cells are selected from cell clusters that are relatively fixed in position and located by a prepattern that is made by interacting gene products [34]. The dynamic situation in the abdomen presents new problems, because in spite of the rapid migration of the sheet of histoblasts, the bristles are eventually very evenly spaced, suggesting that the SOP cells and their descendents adjust their positions, even after they have been identified. This no doubt relates to the fact that bristles generated within a particular epidermal clone in the abdomen (but not the thorax) are frequently displaced from it, sometimes for several cell diameters [35].

### Polarity and bristle development

In our opinion some form of planar polarity is essential to the organisation of epithelia and is likely to operate from the beginning of development; indeed, there is evidence that PCP acts in the early embryo [36-38] and influences the denticles of the larva [3,27] apart from its many effects on the adult. Nevertheless, studies of PCP in the abdomen have concentrated only on cuticular structures such as the bristle shafts (which grow out between 41 h and 48 h APF [9]) and it is not clear what, if any, other outputs of PCP there might be. We see here that the differentiative cell divisions of the bristles [21], the directional migration of the neuronal somata and the outgrowth of axons and dendrites are all oriented, implying the presence of polarising factor(s). Here, we asked whether these factors depend on previously defined PCP genes, such as the Stan system and the Ds system [27].

Previous studies have described the polarity of bristle development in the notum [21,22,39], but the situation in the abdomen is different because the cells' behaviour varies with their position in the A compartment. For example, in the anterior zone of the A compartment, the neuron appears posterior to the sensory cell cluster, whereas in the posterior zone it is found anteriorly. The fifth cell migrates in opposite directions in the two zones. Also, the dendrites differ; in the anterior zone each is connected to its bristle with a U-shape, but, in the posterior zone each dendrite reaches towards the bristle in a straight line. Further, the axons extend in opposing directions in the anterior and posterior zones and even grow laterally in the medial zone. These behaviours are the outcome of several mechanisms that include preferential orientation of the differentiative mitoses, local cell rearrangement, the oriented extension of dendrites and axons and the directional outgrowth of the hairs and bristles. Some of these are consistent over the A compartment (for example, the orientation of bristles – they always point posteriorly) and others not.

Experimentally we used mutations or cell clones to rotate the bristles and see which features were reoriented in a bristle-autonomous way. We found that the shape of dendrites and the way they contact the bristles (with or without a U-shaped turn) was determined entirely by the orientation of the bristle and the relative position of the neuronal soma. In short, if the dendrite can extend from the soma directly into the socket at a point opposite to the shaft (as happens in the wild type at the back of the A compartment where the neuronal soma lies anterior to the bristle), it does so. If not, the dendrite makes a U-turn so that it can enter the bristle from the side opposite to the shaft (as happens in the wild type at the front of the A compartment where the nerve cell lies posterior to the bristle). This suggests that there is only one possible entry direction into the socket of the bristle. It also shows that there is no necessary concordance between the orientation of the bristle, the position of the nerve cell and the paths (in anterior or posterior directions) followed by the neurites, presumably because these latter are determined by different factors.

### Nucleokinesis?

The dendrite appears to connect the neuron to the bristle socket and shaft cells and, subsequently, the neuronal soma moves away as the dendrite is extended. At about the same time the axon grows towards the peripheral abdominal nerve and eventually reaches the CNS. There appears to be a typical growth cone at the axon terminal, which shows filopodia and 'searching movements', so we imagine the axon grows as other axons do. But how is the dendrite extended? We conjecture that this process involves, at least in part, nucleokinesis, meaning that the nucleus moves distally and inside the growing axon. This movement is directed posteriorly in the anterior zone and anteriorly in the posterior zone. However, the evidence for nucleokinesis is only suggestive: we see unstable dilations and swellings always on the axonal side of the neuronal nucleus and, at the same time, elongation of the dendrite, detected as an increase in the distance from the bristle to the nucleus of the neuron. Nucleokinesis has been much discussed in vertebrates, and several mechanisms proposed [25,40-42] but the situation is far from clear. Further studies might now take advantage of *in vivo *imaging of the abdomen and the molecular genetics of *Drosophila*.

The migration of neurons or their precursors was first described during development of the vertebrate brain (for a review, see [43]) and it is essential for normal brain development (see, for example, [44]). In insects, there are but few reports of neural migration. During development of the enteric nervous system of the moth *Manduca sexta *some neurons migrate on the surface of the midgut [45]. Neuronal migration has also been observed in the *Drosophila *embryonic nervous system [46] and in persisting larval sensory neurons [47]. In the nematode *Caenorhabditis elegans *neuronal migration has been studied [48,49].

### Planar cell polarity and neurogenesis

Perturbing the pattern of expression of PCP genes can change the polarity of nearby bristle shafts [13,14] and, as we show here, can alter the relative positions of the neuronal cell bodies. However, the pathways followed by the mechanosensory axons are unaffected by the polarity of the epidermis: even in an extreme case where the polarity of the epidermis is randomised in *ds*^-^*stan*^- ^flies, the orientation of mechanosensory axons is unaffected. Similarly, in the wing it has been observed that the orientation of axon growth is not dependent on the overall polarity [50]. In vertebrate systems it is not known if PCP acts directly on axon orientation, although there are some observations indicating that it might [51-53].

The division of PIIa produces the socket and shaft cell, cells that are consistently aligned with respect to the bristle in all parts of the A compartment. This suggests that the division of PIIa and the disposition of its progeny are dependent on PCP. However, its sister cell (PIIb, generating cells on the neuronal and glial side of the lineage) makes cells that are apparently oriented and positioned somewhat independently of PCP genes: the localisation of the neuronal cell body (behind or in front of the bristle) and the positioning of the fifth cell, as well as its direction of migration, are opposite in the anterior and posterior domains of the A compartment (Figure 4). Thus, while *UAS.fz-*expressing clones suggest that the bristle shafts point normally and consistently from high levels to low levels of Fz, the neuronal processes are oriented and the neuron and associated cells are positioned by other, unknown factors.

This apparent lack of requirement for the Stan system [27], with respect to the pathways followed by axons, is surprising as many experiments have implicated Stan in axon guidance and neurogenesis [52,54-59]. In any case, our observations do not tell us what makes the axons grow in opposite directions in the anterior and posterior zones of the segment. However, as a consequence of this behaviour, the sensory axons never enter the regions close to the segment boundary, ensuring that all the axons emanating from bristles of any one segment are bundled together.

## Conclusion

In each *Drosophila *abdominal segment, the epidermal cells, the bristle shaft and the socket cells are all polarised in the same way. However, the orientation of developing mechanosensory neurons, the sheath cells and the behaviour of the transitory fifth cell differ in ways depending on whether they are located at the front or the back of the segment.

Experiments with genetic mosaics show that some of these polarisations are determined by PCP, while others are not – for example, control by PCP of the orientation of bristle shafts affects both the orientation of mechanosensory dendrites and the relative positions of the somata of neurons. However, our results show that the mechanosensory axons are oriented independently of PCP.

## Materials and methods

### Fly stocks

Unless stated otherwise, Flybase [16] entries of the mutations and transgenes referred to in the text are as follows. *CD2y+*: *Rnor\CD2*^*hs*.*PJ*^. *hs.FLP*: *Scer\FLP1*^*hs*.*PS*^. *his2::DGFP*:*His2Av*^*T*:*Avic*\*GFP*-*S*65*T*^. *UAS.cd8::GFP*: *Mmus\ Cd*8*a*^*Scer*\*UAS*.*T*:*Avic*\*GFP*^. *elav.Gal4*: *elav*^*C*155^. *tub.Gal4*: *Scer\Gal4*^*alphaTub*84*B*.*PL*^. *tub.Gal80*: *Scer\Gal80*^*alphaTub*84*B*.*PL*^*. ds*^-^:*ds*^*UA*071^. *fz*^-^: *fz*^21^. *msi*^-^: *msi*^2^. *Su(H).lacZ*: *Su*(*H*)^*k*07904^. *pros.lacZ*: *pros*^*S*032010^. *UAS.ectoDs *encodes a Ds form that lacks the cytoplasmic domain [27]. *FRT42*: *P{neoFRT}42D*. FRT2A: *P{FRT(w*^*hs*^*)}2A*.

Flies were cultured at 25°C on standard food. A *y w *stock was used as the wild-type strain. All live imaging experiments were carried out on *elav.Gal4 UAS.cd8::GFP *flies. *fz*^- ^clones were produced using *y w hs.FLP; fz*^-^*ri FRT2A*/*CD2y*^+ ^*hs.GFP ri FRT2A*. *tub.Gal4 UAS.ectoDs *clones were obtained using *y w hs.FLP; FRT42 tub.Gal80 CD2y+/FRT42 pwn sha; UAS.ectoDs/tub.Gal4*. Flies described as *ds*^-^*stan*^- ^were actually *y w hs.FLP; ds*^*UA*071^*CD2y*^+ ^*FRT42 stan*^3^*/ds*^*UA*071^*CD2y*^+ ^*FRT42 pwn stan*^*E*59 ^*sha*.

### *In vivo *time-lapse confocal microscopy

White pupae were collected and lined up on double-sided tape on a microscope slide with the dorsal abdomen facing upwards. They were kept at 25°C in Petri dishes containing moist filter paper. Staging time was denoted as hours APF. After removal of the pupal case over the abdomen, pupae were placed in an imaging chamber [60] and a drop of water was added to the pupae. Pupae were imaged from 27 h APF to the pharate adult stage using a BioRad MRC-1024 system laser scanning confocal microscope (Hercules, CA, USA). We used the hemisegment of segment 3 when possible, because its pigmentation is similar in males and females, and its position in the middle of the abdomen makes it easy to study. Approximately 10–15 μm Z-series of confocal images were collapsed using confocal software. Complete adult development took place in the chamber, and adults could hatch from their pupal cuticle, displaying abdomens with a wild-type pattern of bristles. Images were processed using Adobe Photoshop, ImageReady and Illustrator (Adobe Systems, San Jose, CA, USA).

### Clonal inductions and immunohistology

Clones were induced by heat-shocking third instar larvae for 1 h at 34.5°C or 30 minutes at 37°C. Pupae were dissected in phosphate-buffered saline (PBS) in a plastic dish covered with sylgard. The dorsal epithelium was gently washed with PBS. The samples were then fixed in 5.3% formaldehyde (methanol-free, Polysciences Europe, Eppelheim, Germany) in PBS for 20–30 minutes at room temperature. Samples were transferred to 0.5 ml Eppendorf tubes, washed four times with PTX, and then incubated 30 minutes with blocking solution (PTX/1% bovine serum albumin (BSA)). Primary antibodies, 22C10 (mouse, from Hybridoma Bank), anti-GFP (rabbit, from Molecular Probes, Invitrogen Ltd, Paisley, UK), anti-Elav (rat, Molecular Probes), anti-β-galactosidase (rabbit, from Matthew Freeman laboratory, MRC LMB), and anti-prospero (mouse, Molecular Probes) were diluted in PTX/1% BSA 1:40, 1:1,000, 1:500 and 1:1, respectively. Samples were incubated with the primary antibodies for 2 h, and washed with PTX/BSA; incubated with secondary antibodies, FITC or Texas Red-conjugated anti-mouse, anti-rabbit or anti-rat (1:200, Jackson Inmunoresearch Laboratories, West Grove, PA) in PTX/1% BSA. Samples were then washed again, and were kept in Fluoromount-G medium (Southern Biotech, Birmingham, AL, USA) overnight. Abdomens were mounted flat on a slide, with bristles facing upwards, and kept for 2 days at room temperature in the dark, for the fluoromount to solidify. Images were captured with Auto-Montage (Syncroscopy, Cambridge, UK) and processed with Adobe Photoshop.

## Abbreviations

A: Anterior; AP: Anteroposterior; APF: After puparium formation; BSA: Bovine serum albumin; CNS: Central nervous system; P: Posterior; PBS: Phosphate-buffered saline; PCP: Planar cell polarity; SOP: Sensory organ precursor.

## Competing interests

The authors declare that they have no competing interests.

## Authors' contributions

CF was responsible for the vast majority of the work, the observations and experiments, JC supervised the work, especially the genetics, PAL suggested the project and supervised the work and all three authors wrote the paper together and prepared the figures. All authors read and approved the manuscript.

## Supplementary Material

Additional file 1 Some cells expressing *elav *strongly are phagocytosed. Stills from a movie (times given in hours and minutes APF). An individual cell showing few signs of neuronal differentiation (elliptical shape, bipolar protrusions) is followed here. At 30:45 short processes seem to protrude but they are not visible afterwards. A haemocyte appears (33:15, blue arrow) and comes in close contact with the differentiating neuron (33:30). After this transient contact the haemocyte leaves, laden with fluorescent debris, while the neuron now loses its fluorescence and seems to degenerate (33:45). Perhaps we are seeing the death of supernumerary bristle cells or precursors during development, which could influence the final number and/or the spacing of the bristles. Cell death of the larval epidermal cells accompanies the spreading of histoblasts, and these are also eaten up by haemocytes [9]. Click here for file

Additional file 2 Determining position of the neuronal somata relative to their associated bristle. Wild-type and *ds*^-^*stan*^- ^pupae were stained with Elav or 22c10 antibodies and mounted flat. The origin of the coordinate system was fixed at the centre of the socket cuticular structure (considered to represent the position of the adult bristle). The x- and y-axes correspond to the dorso-lateral and AP axes, respectively. The plane of the epithelium corresponds, therefore, to the xy plane. Based on the coordinates of the centre of the socket cuticular structure (0,0) and the centre of the neuronal soma (x_n_, y_n_), we measured the angle between (x_n_, y_n_) and the x-axis. Thus, five categories were defined according to the angle: lateral (ø = 0° ± 5), latero-anterior (0° ± 5 < ø < 90° ± 5), anterior (ø = 90° ± 5), posterior (ø = 270° ± 5), and latero-posterior (270° ± 5 < ø < 360° ± 5). Click here for file
