# Supplementary figures and images for: The abdomen of Drosophila: does planar cell polarity orient the neurons of mechanosensory bristles?
Source: Neural Dev. 2008 Apr 30;3:12. doi: 10.1186/1749-8104-3-12 (PMC2409322; doi:10.1186/1749-8104-3-12)

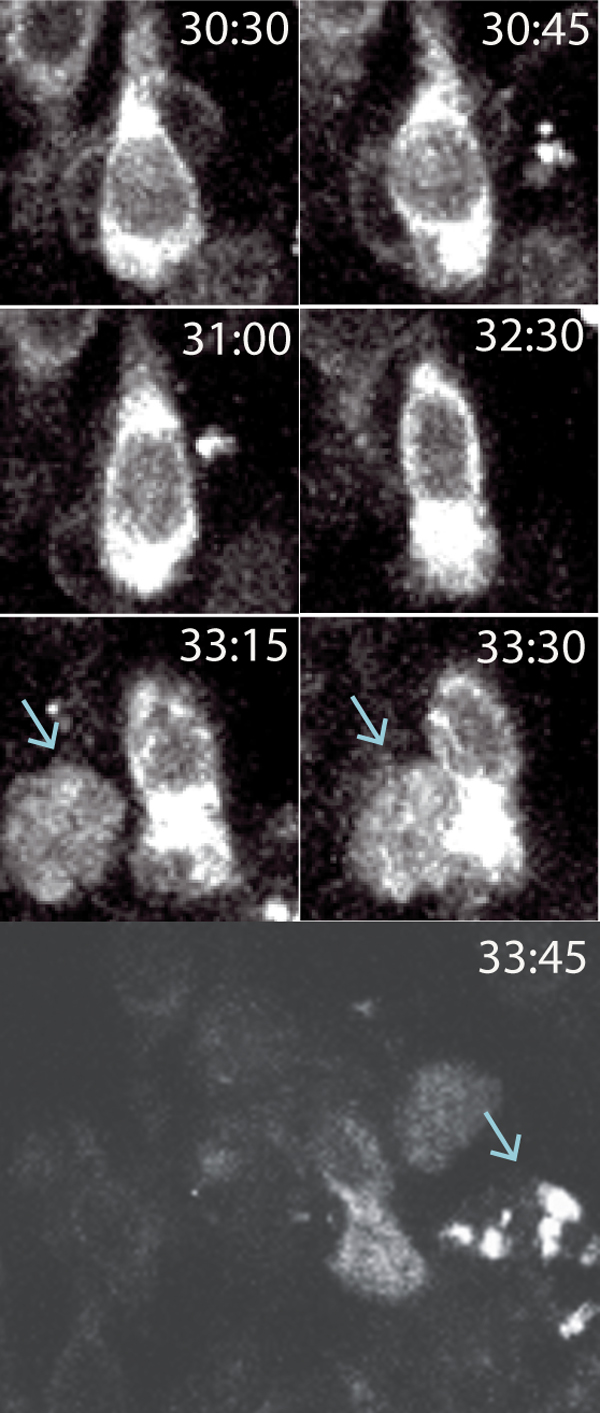

Supplement: Additional file 1 — Some cells expressing elav strongly are phagocytosed. Stills from a movie (times given in hours and minutes APF). An individual cell showing few signs of neuronal differentiation (elliptical shape, bipolar protrusions) is followed here. At 30:45 short processes seem to protrude but they are not visible afterwards. A haemocyte appears (33:15, blue arrow) and comes in close contact with the differentiating neuron (33:30). After this transient contact the haemocyte leaves, laden with fluorescent debris, while the neuron now loses its fluorescence and seems to degenerate (33:45). Perhaps we are seeing the death of supernumerary bristle cells or precursors during development, which could influence the final number and/or the spacing of the bristles. Cell death of the larval epidermal cells accompanies the spreading of histoblasts, and these are also eaten up by haemocytes [9]. [file 1749-8104-3-12-S1.jpeg]

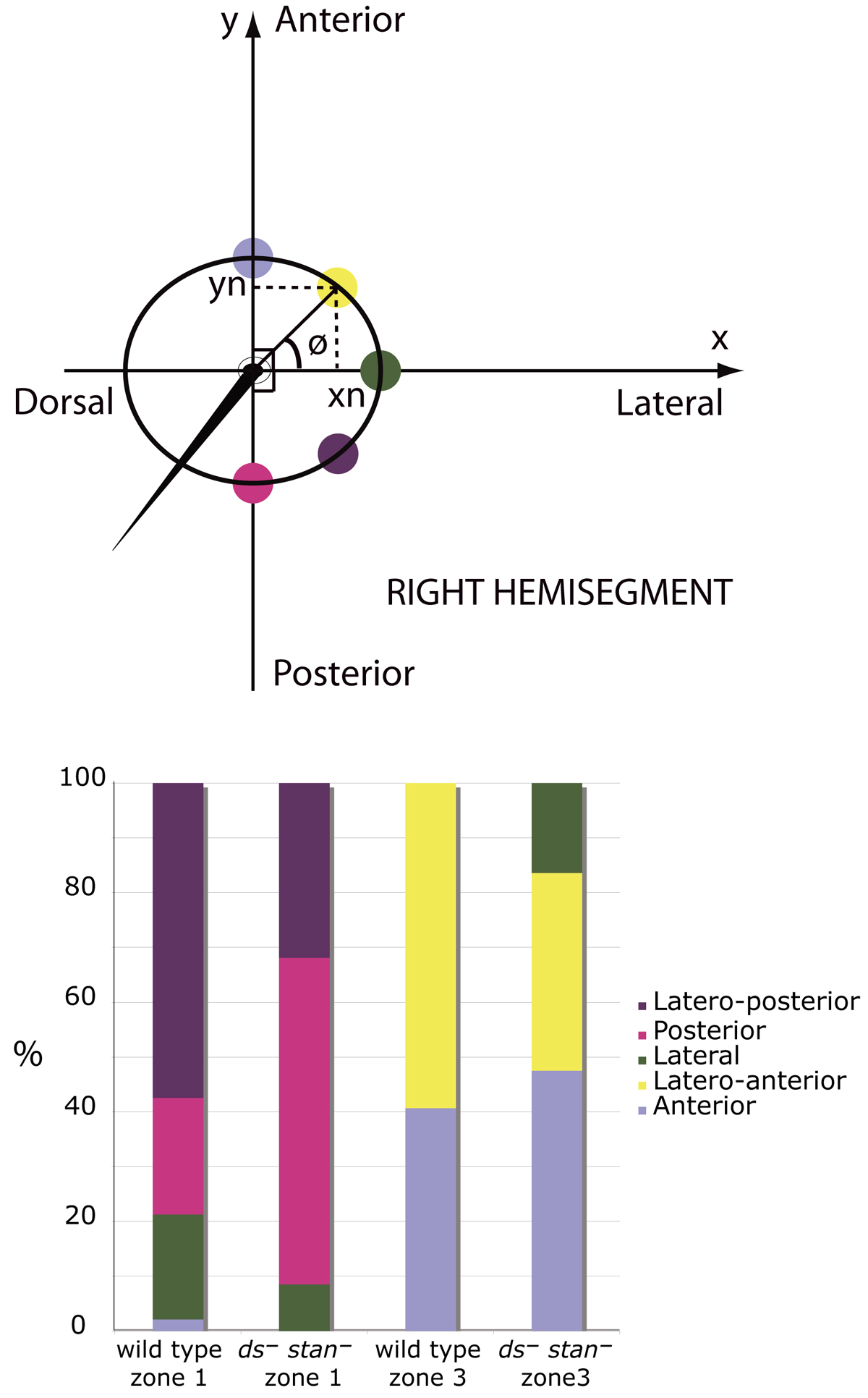

Supplement: Additional file 2 — Determining position of the neuronal somata relative to their associated bristle. Wild-type and ds-stan- pupae were stained with Elav or 22c10 antibodies and mounted flat. The origin of the coordinate system was fixed at the centre of the socket cuticular structure (considered to represent the position of the adult bristle). The x- and y-axes correspond to the dorso-lateral and AP axes, respectively. The plane of the epithelium corresponds, therefore, to the xy plane. Based on the coordinates of the centre of the socket cuticular structure (0,0) and the centre of the neuronal soma (xn, yn), we measured the angle between (xn, yn) and the x-axis. Thus, five categories were defined according to the angle: lateral (ø = 0° ± 5), latero-anterior (0° ± 5 < ø < 90° ± 5), anterior (ø = 90° ± 5), posterior (ø = 270° ± 5), and latero-posterior (270° ± 5 < ø < 360° ± 5). [file 1749-8104-3-12-S2.jpeg]
